# Identification of the *p-*coumaric acid biosynthetic gene cluster in *Kutzneria albida*: insights into the diazotization-dependent deamination pathway

**DOI:** 10.3762/bjoc.20.1

**Published:** 2024-01-02

**Authors:** Seiji Kawai, Akito Yamada, Yohei Katsuyama, Yasuo Ohnishi

**Affiliations:** 1 Department of Biotechnology, Graduate School of Agricultural and Life Sciences, The University of Tokyo, 1-1-1 Yayoi, Bunkyo-ku, Tokyo 113-8657, Japan; 2 Collaborative Research Institute for Innovative Microbiology, The University of Tokyo, Bunkyo-ku, Tokyo 113-8657, Japan

**Keywords:** actinomycetes, avenalumic acid, biosynthesis, *p-*coumaric acid, polyketides

## Abstract

Recently, we identified the biosynthetic gene cluster of avenalumic acid (*ava* cluster) and revealed its entire biosynthetic pathway, resulting in the discovery of a diazotization-dependent deamination pathway. Genome database analysis revealed the presence of more than 100 *ava* cluster-related biosynthetic gene clusters (BGCs) in actinomycetes; however, their functions remained unclear. In this study, we focused on an *ava* cluster-related BGC in *Kutzneria albida* (*cma* cluster), and revealed that it is responsible for *p-*coumaric acid biosynthesis by heterologous expression of the *cma* cluster and in vitro enzyme assays using recombinant Cma proteins. The ATP-dependent diazotase CmaA6 catalyzed the diazotization of both 3-aminocoumaric acid and 3-aminoavenalumic acid using nitrous acid in vitro. In addition, the high efficiency of the CmaA6 reaction enabled us to perform a kinetic analysis of AvaA7, which confirmed that AvaA7 catalyzes the denitrification of 3-diazoavenalumic acid in avenalumic acid biosynthesis. This study deepened our understanding of the highly reducing type II polyketide synthase system as well as the diazotization-dependent deamination pathway for the production of avenalumic acid or *p-*coumaric acid.

## Introduction

The genomes of microorganisms possess diverse biosynthetic gene clusters (BGCs) to produce natural products [1]. In particular, actinomycetes, a major source of bioactive compounds, have numerous BGCs in their genomes, but the majority of them are thought to be silent under laboratory conditions [2]. Because these orphan BGCs are often related to the production of unknown natural products, genome mining has been vigorously used to search for novel compounds in recent years due to the rapid improvement of DNA sequence technologies and computational approaches to analyze BGCs [1,3].

Our research group previously identified the secondary metabolite-specific nitrous acid biosynthetic pathway, named ANS (aspartate-nitrosuccinate), from the study on cremeomycin biosynthesis [4–5]. The ANS pathway is composed of two enzymes, CreE (FAD-dependent monooxygenase) and CreD (lyase), to synthesize nitrous acid from ʟ-aspartate and the nitrous acid is used to synthesize the diazo group of cremeomycin [4]. After the discovery of the ANS pathway, it has been shown that the ANS pathway is involved in the nitrogen–nitrogen (N–N) bond formation in the biosynthesis of several natural products [6–8]. Enzymes that catalyze N–N bond formation by using nitrous acid from the ANS pathway have also been characterized in several recent studies [9–14]. Most of them belong to the adenylate-forming enzyme superfamily (ANL superfamily) and utilize ATP to activate nitrous acid by AMPylation, with the only exception being AzpL in alazopeptin biosynthesis, which is a membrane protein that catalyzes diazotization, presumably in an ATP-independent manner [9–14]. Moreover, the genome database analysis indicated that there are many orphan BGCs containing genes encoding the ANS pathway, which implies that the biosynthesis of many unknown natural products requires nitrous acid derived from the ANS pathway [4].

**Figure 1:**
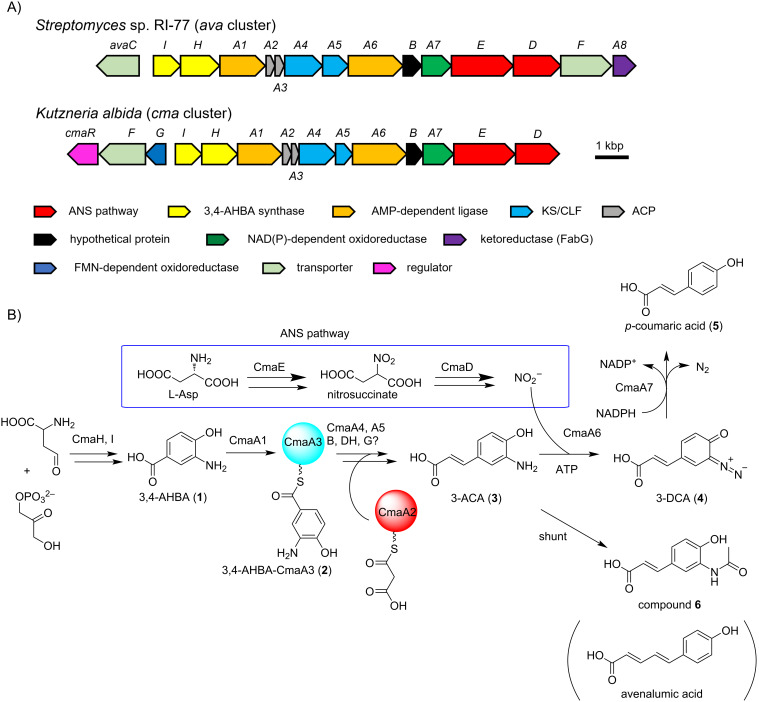
Comparison of *ava* and *cma* clusters and the biosynthetic pathway of *p-*coumaric acid. A) Schematic representation of *ava* and *cma* clusters from *Streptomyces* sp. RI-77 and *Kutzneria albida*, respectively. They harbor similar gene components except for the genes encoding oxidoreductases (*avaA8* and *cmaG*), transporter (*avaC*), and regulator (*cmaR*). B) The proposed biosynthetic pathway of *p-*coumaric acid by the *cma* cluster.

To further understand the role of the ANS pathway in secondary metabolism, we recently identified the BGC for avenalumic acid (*ava* cluster, see the lower right corner of Figure 1B for its structure) by genome mining targeting the ANS pathway in *Streptomyces* sp. RI-77, and revealed its entire biosynthetic pathway [13]. In this pathway, 3-amino-4-hydroxybenzoic acid (3,4-AHBA, **1**), synthesized by AvaH and AvaI, is loaded onto AvaA3 (carrier protein) by AvaA1 (AMP-dependent ligase), resulting in 3,4-AHBA-AvaA3. A highly reducing type II polyketide synthase (PKS) system [15–16] (AvaA2, A4, A5, and A8) then forms a diene moiety using two malonyl units to synthesize 3-aminoavenalumic acid (3-AAA, **7**) from 3,4-AHBA-AvaA3. The amino group of 3-AAA is diazotized by AvaA6 using nitrous acid derived from the ANS pathway (AvaE and AvaD) to form 3-diazoavenalumic acid (3-DAA, **8**), and this diazo group is finally substituted for a hydride derived from NADPH by AvaA7. Interestingly, there are more than 100 BGCs that possess a set of *ava* gene homologs in the genome database, suggesting that these BGCs are responsible for the biosynthesis of avenalumic acid or its derivatives [13].

In this study, we focused on an *ava* cluster-related BGC in *Kutzneria albida* JCM 3240. We showed that this BGC is involved in *p-*coumaric acid biosynthesis by heterologous expression in *Streptomyces albus* J1074 and several in vitro biochemical experiments using recombinant proteins. CmaA6 was shown to catalyze the diazotization of 3-aminocoumaric acid (3-ACA, **3**) and 3-AAA (**7**) with considerably higher efficiency than AvaA6 in avenalumic acid biosynthesis. We also performed kinetic analysis of AvaA7, which catalyzes the denitrification of 3-DAA (**8**) in avenalumic acid biosynthesis, using the highly efficient diazotase CmaA6, for the first time. These results provided new insights into highly reducing type II PKSs and microbial production of *p-*coumaric acid using the ANS pathway, and strengthened our previous proposal for the biosynthesis of avenalumic acid.

## Results

### Bioinformatic analysis of *ava* and *cma* clusters

In our previous study, we found more than 100 BGCs that have a similar gene component to that of the *ava* cluster in the genome database [13]. In this study, to obtain and analyze *ava* cluster-related BGCs, we again searched the genome database for gene clusters that contain (i) genes responsible for 3,4-AHBA synthesis (AvaH and AvaI homologs), (ii) genes encoding a KS-CLF complex (AvaA4 and AvaA5 homologs), and (iii) a gene encoding a diazotase (AvaA6 homolog). As a result, we discovered 134 BGCs and analyzed them using antiSMASH [17] and BiG-SCAPE (Figures S1 and S2 in Supporting Information File 1) [18]. Interestingly, approximately half of them (72 BGCs) do not have *avaE* and *avaD* homologs for the ANS pathway, suggesting three possibilities: (i) *avaE* and *avaD* homologs are encoded apart from the cluster, (ii) the strains have an alternative route to synthesize nitrous acid (as reported for alanosine biosynthesis [19]), and (iii) the strains use exogenous nitrous acid [13]. The BiG-SCAPE analysis divided the BGCs into seven groups (Figures S1 and S2 in Supporting Information File 1) and the *ava* cluster belongs to FAM_00127 (Figure S1). Interestingly, most of the BGCs in FAM_00091, FAM_00111, FAM_00133, and FAM_00125 (approximately 40 BGCs) do not have *avaA8* (FabG-like ketoreductase) and *avaC* (major facilitator superfamily transporter) homologs, but have a *cmaG*-like gene encoding an FMN-dependent oxidoreductase and a *cmaR*-like gene encoding a LysR family transcriptional regulator (Figure 1, Figures S1 and S2 in Supporting Information File 1, and Table 1). We anticipated that they would be a subtype of *ava*-like BGCs and might produce compounds other than avenalumic acid. Among these clusters, we focused on a cluster present in the genome of a rare actinomycete, *Kutzneria albida*, and named it as *cma* cluster because the *cma* cluster was later shown to be responsible for the biosynthesis of *p-*coumaric acid (Figure 1 and Table 1, NCBI accession number CP007155.1).

**Table 1:** Comparison of *cma* and *ava* gene clusters.^a^

Genes in *cma* cluster	Genes in *ava* cluster	Amino acid sequence identity (%)	Predicted function of encoded protein

*cmaR*	–		LysR family transcriptional regulator
*cmaF*	*avaF*	45.9	major facilitator superfamily transporter
*cmaG*	–		FMN-dependent oxidoreductase
–	*avaC*		major facilitator superfamily transporter
*cmaI*	*avaI*	65.4	3,4-AHBA synthase
*cmaH*	*avaH*	66.2	3,4-AHBA synthase
*cmaA1*	*avaA1*	61.4	acyl-ACP ligase
*cmaA2*	*avaA2*	50.5	acyl carrier protein
*cmaA3*	*avaA3*	51.9	acyl carrier protein
*cmaA4*	*avaA4*	62.8	ketosynthase
*cmaA5*	*avaA5*	35.4	chain length factor
*cmaA6*	*avaA6*	65.3	ATP-dependent diazotase
*cmaB*	*avaB*	50.8	hypothetical protein
*cmaA7*	*avaA7*	58.0	NAD(P)-dependent denitrification enzyme
*cmaE*	*avaE*	57.6	aspartate monooxygenase (nitrosuccinate synthase)
*cmaD*	*avaD*	64.7	nitrosuccinate lyase
–	*avaA8*		ketoreductase (FabG)

^a^“–” denotes no counterpart.

### Heterologous expression of *cma* cluster

**Figure 2:**
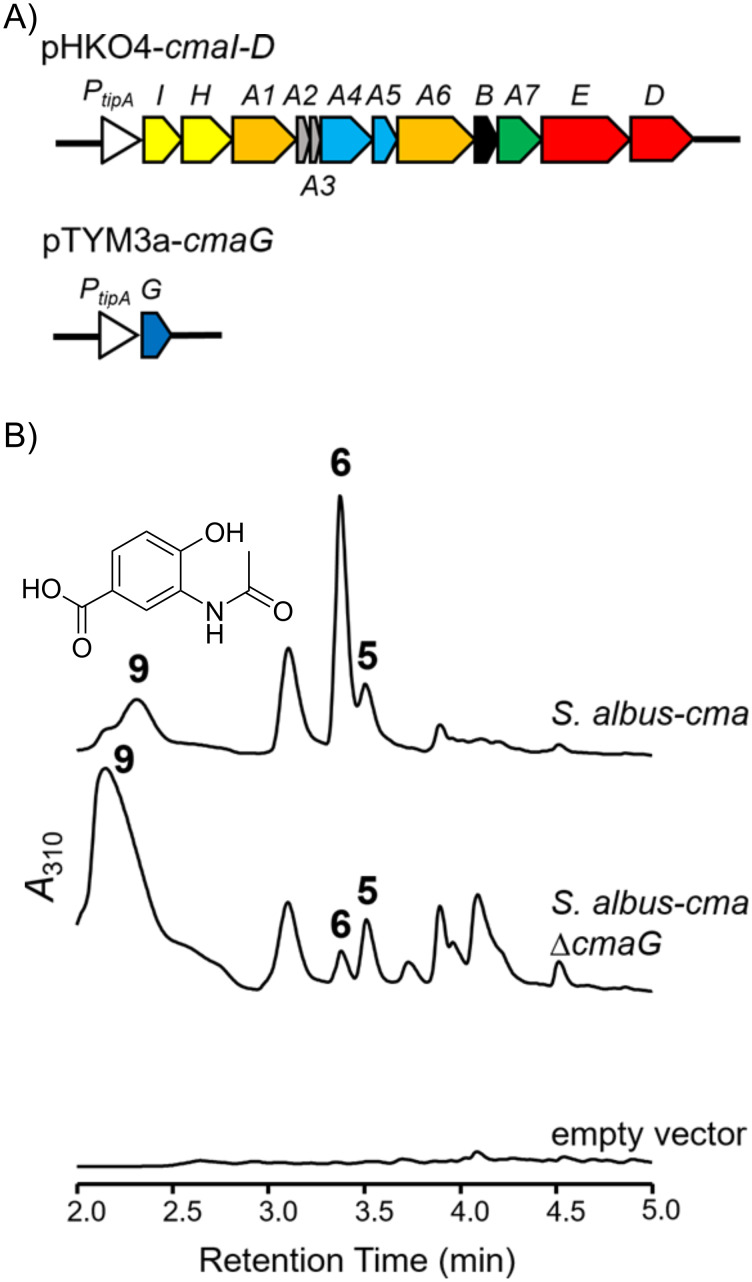
Heterologous expression of the *cma* cluster in *S. albus*. A) Schematic representation of the gene sets on heterologous expression plasmids to construct *S. albus-cma*. The *tipA* promoter was used for gene expression. B) Metabolite analysis of *S. albus-cma*. UV chromatograms at 310 nm are shown. The production yield of **6** was decreased in *S. albus-cma* ∆*cmaG*. Several metabolites produced by *S. albus-cma* and *S. albus-cma* ∆*cmaG* are presumed to be shunt products derived from 3,4-AHBA including *N-*acetyl-3,4-AHBA (**9**). These compounds were not produced by a control strain harboring empty vectors (pHKO4 and pTYM3a).

To examine whether the *cma* cluster is responsible for the biosynthesis of avenalumic acid or another natural product, we performed heterologous expression of the *cma* cluster. Plasmids named pHKO4-*cmaI-D* and pTYM3a-*cmaG* (Figure 2A) were introduced into *S. albus* by conjugation, resulting in *S. albus-cma* [20]. *S. albus-cma* possesses all the *cma* genes, except for the genes encoding a transporter (*cmaF*) and a regulator (*cmaR*), under the control of *tipA* promoter (Figure 2A). When we cultured *S. albus-cma* and analyzed its metabolites by liquid chromatography–mass spectrometry (LC–MS), formation of avenalumic acid was not observed. Instead, production of *p-*coumaric acid (**5**) was detected (Figure 2B and Figure S3A,D in Supporting Information File 1). In addition, *S. albus-cma* produced compound **6**, which showed [M + H]^+^ ion at *m*/*z* = 222, as well as other putative 3,4-AHBA derivatives, among which compound **9** was indicated to be *N-*acetyl-3,4-AHBA (**9**) by its mass and UV spectra (Figure 2B and Figure S3B,C,E,F in Supporting Information File 1). The control strain of *S. albus*, which harbors two empty vectors, did not produce *p-*coumaric acid and compound **6**, suggesting that they are the biosynthetic products of the *cma* cluster (Figure 2B). The yield of compound **6** decreased when *cmaG* was removed from *S. albus-cma*, indicating that CmaG is involved in the production of **6**, although it is not essential (Figure 2B). In addition, the Δc*maG* strain produced a higher amount of compound **9** than *S. albus-cma*. Because compound **9** seems to be a shunt product derived from 3,4-AHBA (**1**), which is the starter substrate of Cma PKS, this result indicated that CmaG is a component of Cma PKS. From NMR analysis and high-resolution (HR)MS analysis ([M + H]^+^ ion at *m*/*z* = 222.0766, which corresponds to C_11_H_12_NO_4_^+^, calcd. 222.0761) of purified **6**, the structure of **6** was determined as *N-*acetyl-3-aminocoumaric acid (Figures S9–S13 and Table S1 in Supporting Information File 1). The production of **6** is consistent with our previous work in which *N-*acetyl-3-aminoavenalumic acid was produced by *S. albus-ava* (an recombinant *S. albus* strain expressing the *ava* cluster for 3-aminoavenalumic acid production) as a shunt product [13]. From these results, we confirmed that the *cma* cluster is responsible for *p-*coumaric acid (**5**) biosynthesis, in which the number of condensations of the malonyl units was one time less than that in the biosynthesis of avenalumic acid.

### In vitro analysis of recombinant Cma proteins

To further understand the biosynthetic machinery encoded by the *cma* cluster, we performed in vitro enzyme analyses using recombinant Cma proteins. First, we obtained recombinant CmaA1, *holo-*CmaA3, and CmaA6 by expressing the corresponding genes in *Escherichia coli* BL21(DE3) (Figure S4 in Supporting Information File 1). Note that *holo-*CmaA3 was obtained by co-expressing with the *sfp* gene to obtain the *holo-*form of CmaA3 [21].

**Figure 3:**
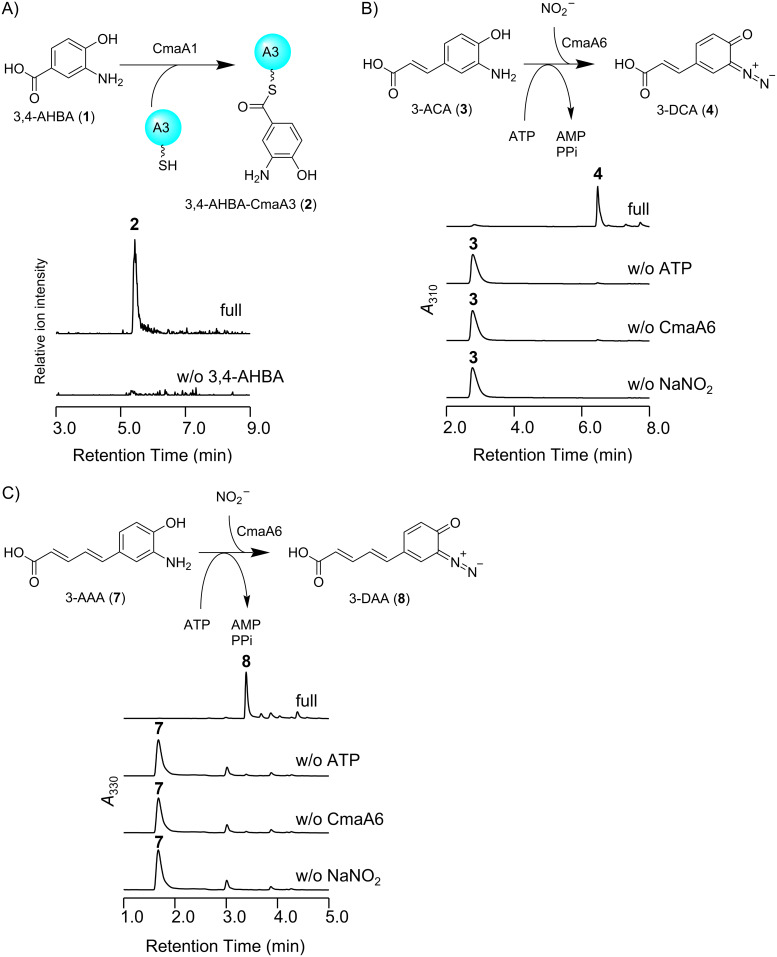
In vitro analysis of Cma proteins. A) In vitro analysis of CmaA1 and CmaA3. Extracted ion chromatograms at *m*/*z* 1087.9, which corresponds to [M + 10H]^10+^ of 3,4-AHBA-CmaA3 (**2**) under positive ion mode, are shown. B, C) In vitro analysis of CmaA6. UV chromatograms at 310 nm and 310 nm are shown. CmaA6 catalyzed diazotization toward 3-ACA (**3**) (B) and 3-AAA (**7**) (C) to synthesize 3-DCA (**4**) and 3-DAA (**8**), respectively, in a high conversion ratio.

We tested whether CmaA1 and *holo-*CmaA3 are involved in the initial reaction in polyketide synthesis similar to AvaA1 and AvaA3; AvaA1 loads 3,4-AHBA (**1**) onto *holo-*AvaA3 using ATP in the biosynthetic pathway of avenalumic acid. As expected, the formation of 3,4-AHBA-CmaA3 (**2**) was verified by LC–MS analysis of the reaction mixture (Figure 3A). Furthermore, we confirmed that AvaA1 can recognize *holo-*CmaA3 to synthesize 3,4-AHBA-CmaA3 (**2**) and that CmaA1 can recognize *holo-*AvaA3 to synthesize 3,4-AHBA-AvaA3 (Figure S5, Supporting Information File 1).

Next, we analyzed the function of CmaA6, which was predicted to catalyze the diazotization of 3-aminocoumaric acid (3-ACA, **3**) in *p-*coumaric acid biosynthesis. As expected, 3-diazocoumaric acid (3-DCA, **4**) was synthesized by CmaA6 in the presence of ATP and sodium nitrite (Figure 3B). Because we supposed that CmaA6 has a promiscuous substrate specificity, we tested whether CmaA6 could also catalyze the diazotization of 3-aminoavenalumic acid (3-AAA, **7**). As expected, CmaA6 also accepted **7** as a substrate to synthesize 3-DAA (**8**) (Figure 3C). Note that the efficiency of diazotization catalyzed by CmaA6 was much higher than that of AvaA6; almost 100% of 3-ACA and 3-AAA were converted to corresponding aromatic diazo compounds **4** and **8**, respectively (Figure 3C).

### Kinetic analysis of AvaA7 catalyzing denitrification of 3-DAA

The high conversion efficiency of 3-AAA (**7**) to 3-DAA (**8**) catalyzed by CmaA6 motivated us to perform the kinetic analysis of AvaA7, which we could not achieve in our previous study. First, we attempted to determine the kinetic values toward NADH and NADPH using compound **8** synthesized by CmaA6. The absorbance at 435 nm (A_435_) exhibited by **8** was measured during the AvaA7-catalyzed reaction (Figure S6D in Supporting Information File 1), and the decrease of A_435_ was tracked to determine the initial velocity of AvaA7. Although the kinetic values toward NADPH could be calculated, the kinetic values toward NADH could not be obtained probably because the *K*_m_ value is very high, suggesting that NADH is not the physiological cofactor of AvaA7 (Figure S6 in Supporting Information File 1 and Table 2). Furthermore, we carried out kinetic analysis of AvaA7 toward **8** in the presence of saturated NADPH (Table 2). The *k*_cat_/*K*_m_ values toward NADH and **8** were reasonable as an NAD(P)-dependent oxidoreductase, suggesting that the denitrification activity of AvaA7 is not an artifact and that AvaA7-catalyzed denitrification is a genuine reaction in bacterial cells (Figure S6 in Supporting Information File 1 and Table 2).

**Table 2:** Kinetic analysis of AvaA7.

	vs NADPH	vs 3-DAA (**8**)

*K*_m_ [µM]	138 ± 10	152 ± 31
*k*_cat_ [min^−1^]	266 ± 2.0	615 ± 55
*k*_cat_/*K*_m_ [min^−1^ µM^−1^]	1.9	4.0

## Discussion

In the present study, we showed that the *cma* cluster is responsible for the production of *p-*coumaric acid using heterologous expression experiments and in vitro enzyme assays. We propose the biosynthetic pathway of *p-*coumaric acid as follows. First, 3,4-AHBA (**1**) synthesized by CmaH and CmaI is loaded onto *holo-*CmaA3 to form 3,4-AHBA-CmaA3 (**2**) by CmaA1. 3-ACA (**3**) is then synthesized from 3,4-AHBA-CmaA3 and malonyl-CmaA2 by the highly reducing type II PKS composed of four Cma proteins (CmaA4, CmaA5, CmaB, and CmaG), a dehydratase from fatty acid synthase, and an unknown thioesterase. CmaB should also stimulate the reaction in an unknown manner as its homolog, AvaB [13]. Next, CmaA6 catalyzes the diazotization of 3-ACA (**3**) using nitrous acid generated by the ANS pathway (CmaE and CmaD) to form 3-DCA (**4**). Finally, CmaA7 catalyzes the denitrification of 3-DCA (**4**) using NADPH to synthesize *p-*coumaric acid (**5**).

Although we have not yet investigated the activity of CmaG (a putative FMN-dependent oxidoreductase), it is expected to catalyze the reduction of the keto group of an β-ketoacyl intermediate, similar to the ketoreductase of fatty acid synthase (FAS) and highly reducing type II PKS. This hypothesis is supported by the fact that *cmaG* exists in the *cma* cluster instead of *avaA8*, which encodes a putative FabG-like ketoreductase, and by the observation that the yield of compound **6** decreased considerably when *cmaG* was removed from the heterologous expression system (Figure 1A and Figure 2B, and Figures S1 and S2 in Supporting Information File 1). It should be emphasized that the production of **5** and **6** was not completely abolished in *S. albus-cma* ∆*cmaG*. This is because FAS’s ketoreductase probably interacts with the Cma system in the absence of CmaG.

The loading of 3,4-AHBA onto ACP (CmaA3), which is the initial reaction of Cma PKS, is catalyzed by CmaA1, as shown by in vitro analysis. Interestingly, both *ava* and *cma* clusters have two ACP genes. These ACPs are expected to have different roles in each biosynthetic pathway; AvaA3 and CmaA3 carry the starter substrate (3,4-AHBA) and AvaA2 and CmaA2 carry the extender unit (malonyl moiety) [13]. CmaA2 and CmaA3 showed relatively high amino acid sequence similarity to AvaA2 and AvaA3, respectively (≈50% identity; see Table S2 in Supporting Information File 1). In contrast, low similarities were observed between CmaA2 and AvaA3, and between CmaA3 and AvaA2 (Table S2, Supporting Information File 1). Because AvaA1 was shown not to recognize AvaA2, but to recognize AvaA3 in vitro, the homologs of these proteins (CmaA1, CmaA2, and CmaA3) are expected to have the same features. The mechanism by which AvaA1 and CmaA1 distinguish between two different ACPs (AvaA2 and AvaA3 for AvaA1 and CmaA2 and CmaA3 for CmaA1) is a matter of interest. The fact that AvaA1 and CmaA1 recognized CmaA3 and AvaA3, respectively, as partner ACP suggests that there are some important residues conserved between AvaA3 and CmaA3 for the ligase–ACP interaction. Indeed, two amino acid residues (Trp38 and His41 of CmaA3), which seem to be important for the formation of the CmaA1–CmaA3 complex in the structural model predicted by AlphaFold2 [22–23], are conserved in AvaA3 and CmaA3, but not in AvaA2 and CmaA2 (Figure S7 in Supporting Information File 1). Further analysis of these systems would provide important insights into how highly reducing type II PKSs control starter substrate incorporation.

The only difference between avenalumic acid and *p-*coumaric acid is the chain length. The CLF CmaA5 shows 35.4% identity to AvaA5, which is the lowest similarity score among Ava and Cma protein pairs (Table 1). This is reasonable because CLF is involved in the chain length control of type II PKSs [24–25]. Phylogenetic analysis of CLFs encoded in the homolog gene clusters of *ava* cluster-related BGCs indicated that they can be divided into three large clades (Figure S8 in Supporting Information File 1). AvaA5 and CmaA6 belong to different clades, and the proteins belonging to the third clade have a low sequence similarity to both AvaA5 and CmaA6. Thus, the *ava* cluster-related BGCs that encode a CLF belonging to the third clade likely produce analog compounds with different chain lengths.

*p-*Coumaric acid is an intermediate in flavonoid biosynthesis and is much more ubiquitous than avenalumic acid. In general, *p-*coumaric acid is synthesized by phenylalanine ammonia-lyase and cinnamate 4-hydroxylase from phenylalanine or by a tyrosine ammonia-lyase from tyrosine [26]. However, the Cma system synthesizes *p-*coumaric acid through a completely different pathway, which requires at least 12 enzymes and two carrier proteins if two primary metabolites (dihydroxyacetone phosphate and aspartate-4-semialdehyde) are considered as starting materials. Thus, the Cma system appears to be more complicated than the general *p-*coumaric acid synthesis. Therefore, it is interesting to understand why actinomycetes synthesize *p-*coumaric acid using such a specialized biosynthetic pathway including diazotization and denitrification. It should be noted that only three ammonia-lyases for *p-*coumaric acid biosynthesis have been characterized in actinomycetes [27–28]. The Cma system may have some advantages over the general *p-*coumaric acid biosynthetic pathways when actinomycetes evolutionally develop the *p-*coumaric acid biosynthetic pathway in secondary metabolism.

We identified CmaA6 as an ATP-dependent 3-ACA diazotase that showed a very high conversion efficiency. CmaA6 also catalyzed the diazotization of 3-AAA to synthesize 3-DAA with a much higher conversion efficiency than AvaA6 in avenalumic acid biosynthesis; CmaA6 converted almost all 3-ACA (**3**) to 3-DCA (**4**), whereas AvaA6 converted approximately 10% of 3-ACA (**3**) to 3-DCA (**4**) within 1 h. This property is probably due to the high stability of CmaA6. The high activity of CmaA6 makes it a useful tool for biochemical experiments and chemoenzymatic synthesis. Indeed, the discovery of CmaA6 allowed us to analyze the kinetics of the denitrification reaction catalyzed by AvaA7, which had been difficult to achieve in our previous studies because of the low enzymatic activity of AvaA6. The kinetic values for AvaA7 are in a reasonable range for an enzyme of this family (e.g., some NADPH-dependent sugar epimerases reported to have *k*_cat_/*K*_m_ values of 0.79 and 0.86 min^−1^ µM^−1^) and strongly support our previous observation that AvaA7 showed a preference for NADPH as a cofactor [13,29]. In addition, CmaA6 could be an attractive target for understanding the reaction mechanism of ATP-dependent diazotase. CmaA6 could also be an ancestor for generating useful biocatalysts to synthesize diazo group-containing compounds through directed evolution.

Finally, this study is important because of the success of genome mining for BGCs in a rare actinomycete, *K. albida*. Although 47 BGCs were indicated in the *K. albida* genome by the antiSMASH analysis, only aculeximycin was reported as a natural product produced by *K. albida* [30]. Although rare actinomycetes have been expected to be a source of novel natural products, reports of natural product isolation from rare actinomycetes are limited because of the difficulty in cultivation and genetic manipulation. This study demonstrates that heterologous expression of BGCs combined with promoter swapping is a powerful tool for the discovery of new natural products from rare actinomycetes.

## Conclusion

In summary, we identified a *cma* gene cluster for *p-*coumaric acid biosynthesis in the *K. albida* genome. We demonstrated the following two biosynthetic reactions in the Cma system using in vitro enzyme assays. (i) CmaA1 loads 3,4-AHBA (**1**) onto *holo-*CmaA3 using ATP. (ii) CmaA6 catalyzes the diazotization of 3-ACA (**3**) to produce 3-DCA (**4**) in the presence of ATP and sodium nitrite. By analogy with the Ava system, we concluded that the Cma system biosynthesizes *p-*coumaric acid via diazotization-dependent deamination. The diazotase CmaA6 had a high catalytic activity, which enabled us to determine the kinetics of the denitrification reaction catalyzed by AvaA7. Furthermore, careful comparison between *ava* and *cma* clusters provided insights into (i) the mechanism of protein–protein interaction between the carrier protein and AMP-dependent ligase and (ii) the chain length control of highly reducing type II PKSs.

## Experimental

### Strains, chemicals, and enzymes

*E. coli* JM109 was used for DNA manipulation, and *E. coli* BL21(DE3) was used for expressing recombinant proteins. *E. coli* S17-1 was used for conjugation. *Streptomyces albus* J1074 was used for heterologous expression. *Kutzneria albida* JCM 3240 was purchased from the Japan Collection of Microorganisms. Enzymes used for DNA manipulation, including polymerase and restriction enzymes, were purchased from TaKaRa Bio Inc. (Shiga, Japan). Primers were purchased from Thermo Fisher Scientific (Waltham, MA, USA). Chemicals for the enzymatic assay were purchased from Sigma-Aldrich (St. Louis, MO, USA). Chemicals for organic synthesis were purchased from Tokyo Chemical Industry (Tokyo, Japan). 3-Aminocoumaric acid (**3**) and 3-aminoavenalumic acid (**7**) were synthesized in our previous study [13].

### Construction of heterologous expression vectors

First, pHKO4-*cmaI-D* was constructed by cloning the *cmaI*-*H-A1-A2-A3-A4-A5-A6-B-A7-E-D* operon (which was amplified by PCR using *K. albida* genomic DNA as the template) into the NdeI and HindIII sites of pHKO4 using In-Fusion (TaKaRa Bio Inc.) [20]. pTYM3a-*cmaG* was constructed by cloning *cmaG* (which was amplified by PCR using *K. albida* genomic DNA as the template) into the NdeI and XbaI sites of pTYM3a using In-Fusion (TaKaRa Bio Inc.). The primers used for plasmid construction are listed in Table S3 of Supporting Information File 1.

### Heterologous expression of the *cma* cluster in *S. albus*

pHKO4*-cmaI-D* and pTYM3a-*cmaG* were introduced into *S. albus* to construct *S. albus-cma* in the same way to construct *S. albus-ava* described in our previous study [13].

*S. albus-cma* was inoculated into 10 mL of Tryptone Soya Broth (TSB) medium with kanamycin (50 mg/L) and incubated with shaking (300 rpm) at 30 °C for 2 days. One milliliter of this preculture medium was inoculated into 100 mL of Waksman medium (5 g/L Bacto^TM^ peptone, 5 g/L Bacto^TM^ yeast extract, 20 g/L glucose, 5 g/L meat extract, 5 g/L NaCl, and 3 g/L CaCO_3_) with kanamycin (50 mg/L), and incubated with shaking (120 rpm) at 30 °C for 2 days. Thiostrepton (15 mg/L) was then added to the culture, and the culture was continued for another 1 day.

After incubation, brine (0.5 mL) was added to 5 mL of the culture, and the metabolites were extracted with 5 mL of ethyl acetate after adjusting the pH to approximately 4 by adding 6 M HCl. The ethyl acetate layer was collected, and it was washed with an equal volume of distilled water to remove the compounds that can be dissolved in water. The ethyl acetate layer was then collected and evaporated completely in vacuo. The residual materials were dissolved in 200 µL of methanol. The obtained samples were analyzed by liquid chromatography–electrospray ionization mass spectrometry (LC–ESIMS) using an LC-2040C 3D Plus system (Shimazu Corp., Kyoto, Japan) equipped with a COSMOCORE 2.6C18 Packed column (2.1 mm ID × 100 mm, Nacalai Tesque) coupled with a model LCMS-8040 liquid chromatography–mass spectrometer (LC–MS) (Shimazu Corp.). The compounds were eluted with a linear gradient of water/acetonitrile containing 0.1% formic acid.

### Isolation and structural determination of compound **6**

*S. albus-cma* was inoculated into 10 mL of TSB medium with kanamycin (50 mg/L) and incubated with shaking (300 rpm) at 30 °C for 2 days. The whole culture was transferred into 1 L of Waksman medium with kanamycin (50 mg/L) and incubated with shaking (120 rpm) at 30 °C for 2 days. Then, thiostrepton (15 mg/L) was added to the culture, and the culture was continued for another 1 day. The pH was adjusted to 4 by adding 6 M HCl to the culture, and the metabolites were extracted by ethyl acetate. After the ethyl acetate was evaporated, the metabolites were absorbed for 5.0 g silica gel 60 (0.040–0.063 mm, Merck Millipore, Burlington, MA, USA). The silica gel holding the metabolites was applied to a normal-phase medium-pressure liquid chromatography system (MPLC, Shoko Scientific, Kanagawa, Japan) equipped with a silica column (Purif-Pack, Shoko Scientific), and the metabolites were eluted with a linear gradient of chloroform/methanol. Fractions containing compound **6** were concentrated by evaporation. The residual materials were desorbed in 1 mL DMSO and applied to a reversed-phase high-performance liquid chromatography (HPLC, Shimadzu Corp.) equipped with a COSMOCORE Packed column 5C18-AR-II (10 mm ID × 250 mm, Nacalai Tesque), and the metabolites were eluted with a linear gradient of water/acetonitrile containing 0.1% formic acid. Fractions containing compound **6** were concentrated by evaporation. Compound **6** was then desorbed in DMSO-*d*_6,_ and the structure was determined by the JNM-A600 NMR system (JEOL, Tokyo, Japan) (Table S1 and Figures S9–S13 in Supporting Information File 1).

### Production and purification of recombinant proteins

pColdI-*cmaA1*, pColdI-*cmaA3*, and pColdI-*cmaA6* were constructed by cloning each gene (which was amplified by PCR using pHKO4-*cmaI-D* as a template) into the NdeI and XhoI sites of pColdI using In-Fusion (TaKaRa Bio Inc.). The primers used for plasmid construction are listed in Table S4 of Supporting Information File 1.

Each protein expression plasmid was introduced into *E. coli* BL21(DE3). To obtain *holo-*CmaA3, pACYC-*sfp* [21] was co-introduced with pColdI-*cmaA3* into *E. coli* BL21(DE3). The obtained strain was cultured in 1 L of Terrific broth (24 g/L yeast extract, 12 g/L tryptone, 0.8% glycerol, 0.94% K_2_HPO_4_, and 0.22% KH_2_PO_4_) with ampicillin and chloramphenicol (if necessary for *sfp* expression) and incubated with shaking (150 rpm) at 37 °C until the OD_600_ reached 0.8. After the culture was cooled on ice for 30 min, isopropyl β-ᴅ-thiogalactopyranoside (IPTG) was added to the culture (for CmaA1 and CmaA3 expression, final concentration was 100 µM; for CmaA6 expression, IPTG was not added) and incubated with shaking (150 rpm) at 15 °C for 20 h. Cells were harvested by centrifugation and suspended in lysis buffer (20 mM HEPES-NaOH, 10% glycerol, and 200 mM NaCl_2_; pH 8.0). The cells were disrupted by sonication on ice, and cell debris was removed by centrifugation. The recombinant protein was purified using His60 Ni Superflow Resin (TaKaRa Bio Inc.). The protein was eluted using a stepwise gradient of imidazole in lysis buffer (20–500 mM imidazole). The buffer was replaced with lysis buffer using an Amicon Ultra centrifugal filter with a suitable molecular mass cutoff (Merck Millipore).

Recombinant AvaA1 and AvaA3 obtained in our previous study [13] were used in the current experiment.

### In vitro analysis of CmaA1, CmaA3, AvaA1, and AvaA3

A reaction mixture (50 µL) containing 4 µM CmaA1 or AvaA1, 40 µM CmaA3 or AvaA3, 2.5 mM 3,4-AHBA, 1 mM ATP, 5 mM MgCl_2_, 20 mM HEPES-NaOH (pH 8.0), 10% glycerol, and 200 mM NaCl was prepared and incubated at 30 °C for 1 h. After centrifugation, the supernatant was analyzed using LC–MS equipped with a BioResolve RP mAb polyphenyl column (2.1 mm ID × 100 mm, Waters, Milford, MA, USA). The compounds were eluted using a linear gradient of water/acetonitrile containing 0.1% formic acid.

### In vitro analysis of CmaA6

A reaction mixture (50 µL) containing 5 µM CmaA6, 0.5 mM 3-ACA (**3**), 1 mM ATP, 5 mM NaNO_2_, 5 mM MgCl_2_, 20 mM HEPES-NaOH (pH 8.0), 10% glycerol, and 200 mM NaCl was prepared and incubated at 30 °C for 1 h. The reaction was quenched by adding 50 µL of methanol. After centrifugation, the supernatant was analyzed by LC–MS equipped with a COSMOCORE 2.6Pbr Packed column (2.0 mm ID × 100 mm, Nacalai Tesque). The compounds were eluted with a linear gradient of water/acetonitrile containing 0.1% formic acid.

A reaction mixture (50 µL) containing 5 µM CmaA6, 0.5 mM 3-AAA (**7**), 1 mM ATP, 5 mM NaNO_2_, 5 mM MgCl_2_, 20 mM HEPES-NaOH (pH 8.0), 10% glycerol, and 200 mM NaCl was prepared and incubated at 30 °C for 1 h. The reaction was quenched by adding 50 µL methanol. After centrifugation, the supernatant was analyzed by LC–MS, following the same method used for "Heterologous expression of *cma* cluster using *S. albus*."

### Kinetic analysis of AvaA7

To measure the kinetic parameters of NADPH or NADH, the reaction mixture (90 µL) containing 10 µM CmaA6, 1.0 mM 3-AAA (**7**), 2 mM ATP, 5 mM NaNO_2_, 2.5 mM MgCl_2_, 20 mM HEPES-NaOH (pH 8.0), 10% glycerol, and 200 mM NaCl was prepared and incubated at 30 °C for 1 h. Then, 5.0 µL of 4.0 µM AvaA7 (final concentration: 0.2 µM) and 5.0 µM of different concentrations of NADPH or NADH (final concentration: 25–800 µM) were added to the reaction mixture (final reaction volume: 100 µL). The initial velocity of the reaction catalyzed by AvaA7 was estimated by monitoring the decrease in absorbance at 435 nm for 3-DAA using a SpectraMax M2 microplate reader (Molecular Devices, San Jose, CA, USA). The kinetic parameters (*V*_max_, *k*_cat_, and *K*_m_) were calculated by fitting the substrate concentration [S]–initial velocity (*v*) plot using the equation *v* = *V*_max_ × [S] / (*K*_m_ + [S]).

To measure the kinetic parameters of 3-DAA, the reaction mixture (90 µL) containing 10 µM CmaA6, 1.0 mM 3-AAA (**7**), 2 mM ATP, 5 mM NaNO_2_, 2.5 mM MgCl_2_, 20 mM HEPES-NaOH (pH 8.0), 10% glycerol, and 200 mM NaCl was prepared and incubated at 30 °C for 1 h. Then, the concentration of 3-DAA (**8**) in the reaction mixture was calculated by measuring the absorbance at 435 nm for 3-DAA (**8**) using a SpectraMax M2 microplate reader. The initial velocity of the reaction catalyzed by AvaA7 was calculated following the same method as mentioned above, after diluting the reaction mixture to a final concentration of 25 to 400 µM and adding AvaA7 (final concentration: 0.2 µM) and NADPH (final concentration: 800 µM).

## Supporting Information

File 1 Additional experimental data and NMR spectra.

## Data Availability

All data that supports the findings of this study is available in the published article and/or the supporting information to this article.
