# Supplementary material for: Identification of the p-coumaric acid biosynthetic gene cluster in Kutzneria albida: insights into the diazotization-dependent deamination pathway
Source: Beilstein J Org Chem. 2024 Jan 2;20:1–11. doi: 10.3762/bjoc.20.1 (PMC10777205; doi:10.3762/bjoc.20.1)
Supplement: File 1 — Additional experimental data and NMR spectra. [file Beilstein_J_Org_Chem-20-01-s001.pdf]

BEILSTEIN JOURNAL OF ORGANIC CHEMISTRY

Supporting Information

for

Identification of the *p*-coumaric acid biosynthetic gene cluster in *Kutzneria albida*: insights into the diazotization-dependent deamination pathway

Seiji Kawai, Akito Yamada, Yohei Katsuyama and Yasuo Ohnishi

Beilstein J. Org. Chem. **2024,** *20,* 1–11. doi:10.3762/bjoc.20.1

Additional experimental data and NMR spectra

License and Terms: This is a supporting information file under the terms of the Creative Commons Attribution License (https://creativecommons.org/licenses/by/4.0). Please note that the reuse, redistribution and reproduction in particular requires that the author(s) and source are credited and that individual graphics may be subject to special legal provisions.
The license is subject to the *Beilstein Journal of Organic Chemistry* terms and conditions: (https://www.beilstein-journals.org/bjoc/terms)

Table of contents

Table S1. ^{1}H and ^{13}C NMR data for compound **6**.
Table S2. Amino acid identity between ACPs.
Table S3. Primers used for the construction of heterologous expression plasmids.
Table S4. Primers used for the construction of recombinant protein production plasmids.

Figure S1. The BiG-SCAPE analysis of *ava*-like clusters (Part 1).
Figure S2. The BiG-SCAPE analysis of *ava*-like clusters (Part 2).
Figure S3. LC-MS analysis of the metabolites of *S. albus-cma* which produces compounds **5** and **6** and other 3,4-AHBA derivatives.
Figure S4. SDS-PAGE analysis of the recombinant proteins used in this study.
Figure S5. *In vitro* analysis of CmaA1, *holo*-CmaA3, AvaA1, and *holo*-AvaA3.
Figure S6. Kinetic analysis of AvaA7, fitted with the Michaelis-Menten equation.
Figure S7. Insights into the mechanism of partner ACP (CmaA3) recognition mechanism by CmaA1.
Figure S8. Phylogenetic analysis of CLFs encoded by the by *ava*-related BGCs in the database.
Figure S9. ^{1}H NMR spectrum of compound **6**.
Figure S10. ^{13}C NMR spectrum of compound **6**.
Figure S11. ^{1}H-^{1}H COSY spectrum of compound **6**.
Figure S12. ^{1}H-^{13}C HMQC spectrum of compound **6**.
Figure S13. ^{1}H-^{13}C HMBC spectrum of compound **6**

Supplementary tables

Table S1. ^{1}H and ^{13}C NMR data for compound **6**. The spectra were recorded in DMSO-d_6. The solvent peak was used as an internal standard (δ_C 39.51, δ_H 2.50).

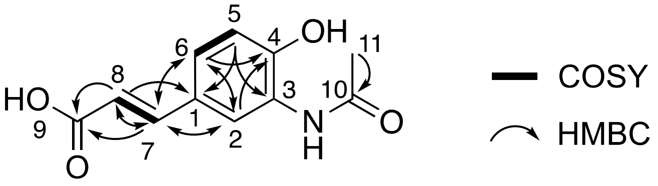

Position	δ_C	δ_H (multi., J in Hz)
1	126.8	–
2	121.9	8.01 (s, 1H)
3	125.1	–
4	150.1	–
5	115.8	6.84 (d, 8.7, 1H)
6	125.4	7.25 (d, 8.7, 1H)
7	144.2	7.41 (d, 15.6, 1H)
8	115.8	6.17 (d, 15.6, 1H)
9	167.8	–
10	169.1	–
11	23.7	2.06 (s, 3H)

Table S2. Amino acid identity between ACPs

	AvaA2	AvaA3	CmaA2	CmaA3
AvaA2	-	29.0%	50.5%	24.4%
AvaA3	29.0%	-	25.3	51.9%
CmaA2	50.5%	25.3%	-	22.9%
CmaA3	24.4%	51.9%	22.9%	-

Table S3. Primers used for the construction of heterologous expression plasmids.

name	sequence	description
cmaI-D F	5'-AAGGGAGCGGACATATGGCTGACACCGGGAAAGCG-3'	NdeI site is underlined
cmaI-D R	5'-GGTCCTGCCCAAGCTTTCAGGTGCTGAGCCGGTACC-3'	HindIII site is underlined
cmaG F	5'-AAGGGAGCGGACATATGTCCTCAGATCGGAGAGG-3'	NdeI site is underlined
cmaG R	5'-GCAGGTCGACTCTAGATTACCGCGCGGGTGCCAGTGC-3'	XbaI site is underlined

Table S4. Primers used for the construction of recombinant protein production plasmids.

name	sequence	description
cmaA1 F	5'-TCGAAGGTAGGCATATGCGGCTGGTGGAGGACCT-3'	NdeI site is underlined
cmaA1 R	5'-ATTCGGATCCCTCGAGTCAACTGATCGCCGCGCGCA-3'	XhoI site is underlined
cmaA3 F	5'-TCGAAGGTAGGCATATGACCACCGAGCAGGTGCGC-3'	NdeI site is underlined
cmaA3 R	5'-ATTCGGATCCCTCGAGTCATCGCACCGCCCCGGAGT-3'	XhoI site is underlined
cmaA6 F	5'-TCGAAGGTAGGCATATGATCACCAAGGAAGAACGC-3'	NdeI site is underlined
cmaA6 R	5'-ATTCGGATCCCTCGAGTCACCCCTGGTCGGCGCGGG-3'	XhoI site is underlined

Supplementary figures

FAM_00108

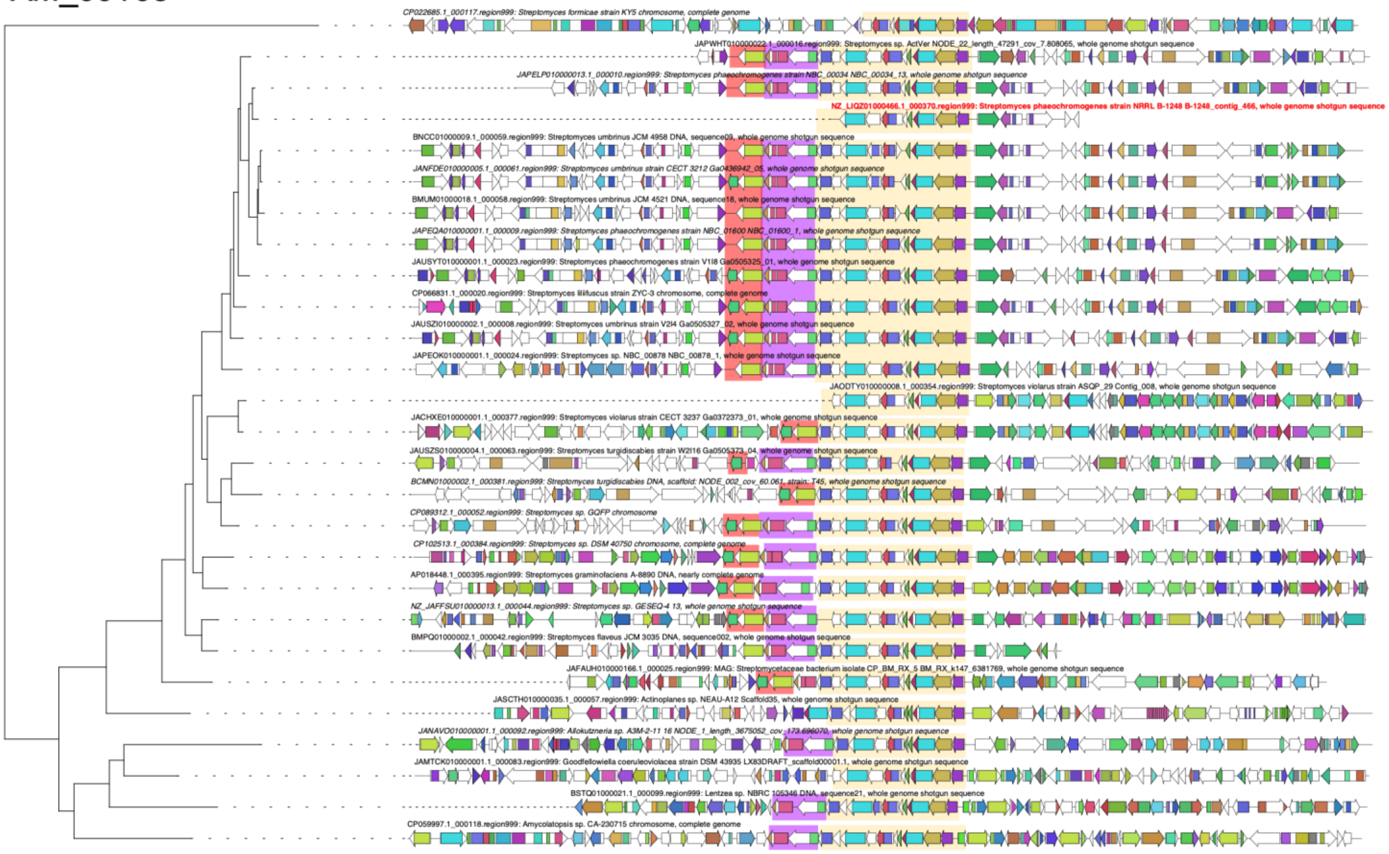

FAM_00132

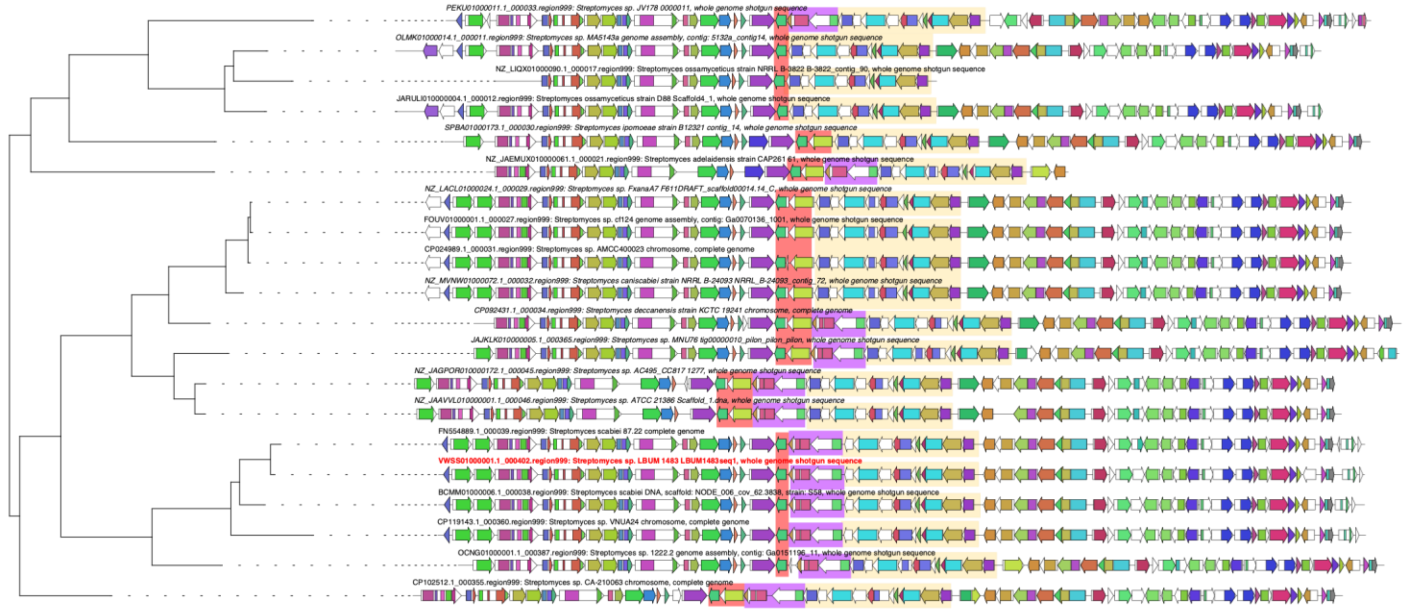

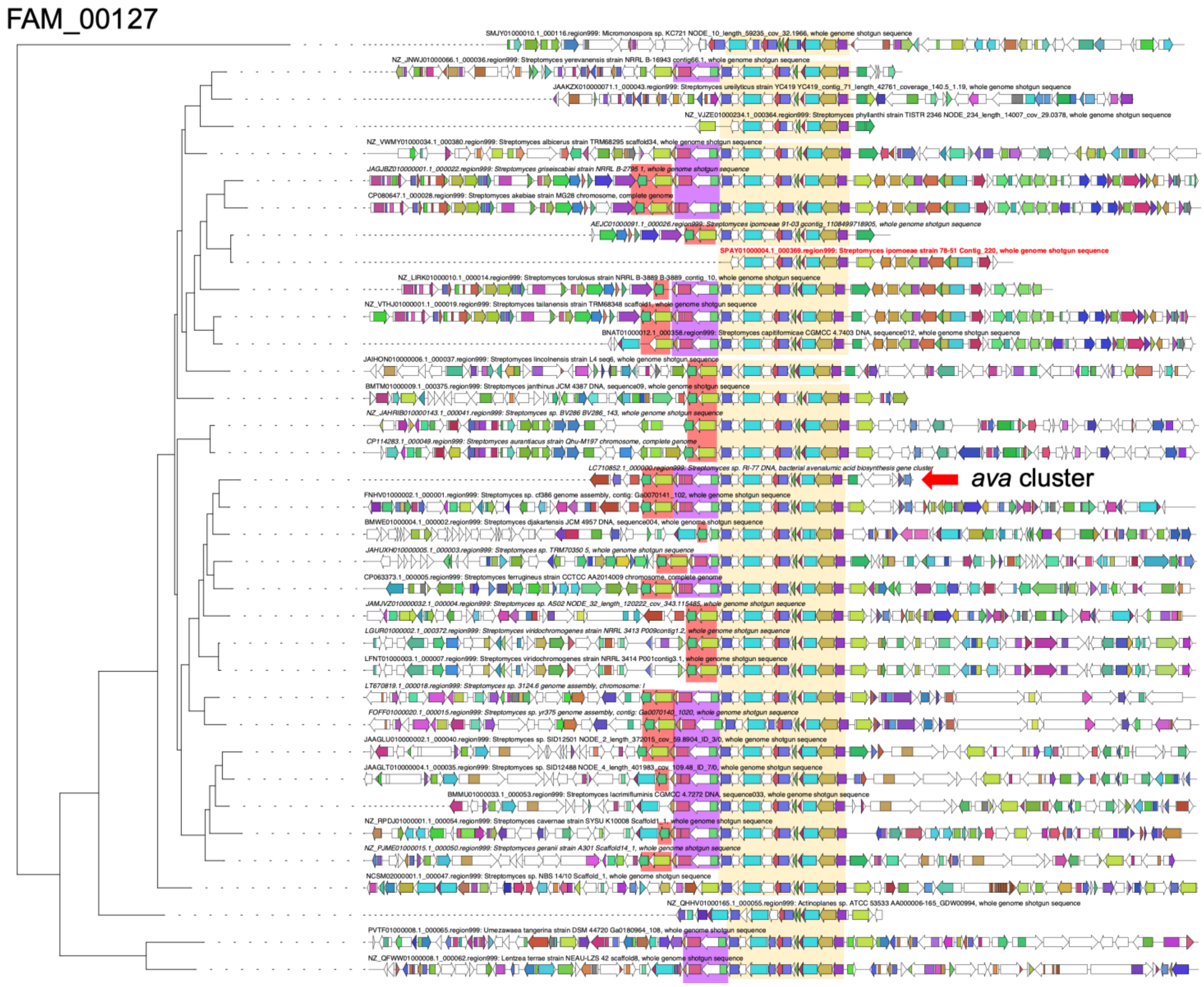

Figure S1. The BiG-SCAPE analysis of *ava*-like clusters (Part 1). The clusters belong to FAM_00127, FAM_00108, and FAM_00132 are shown. *avaI*, *H*, *A1, A2, A3, A4, A5*, *A6*, *B*, and *A7* homologs are highlighted in yellow. *avaE* and *avaD* homologs are highlighted in purple. *avaF* and *avaA8* homologs are highlighted in red. The colors of Pfam domains are automatically generated by BiG-SCAPE.

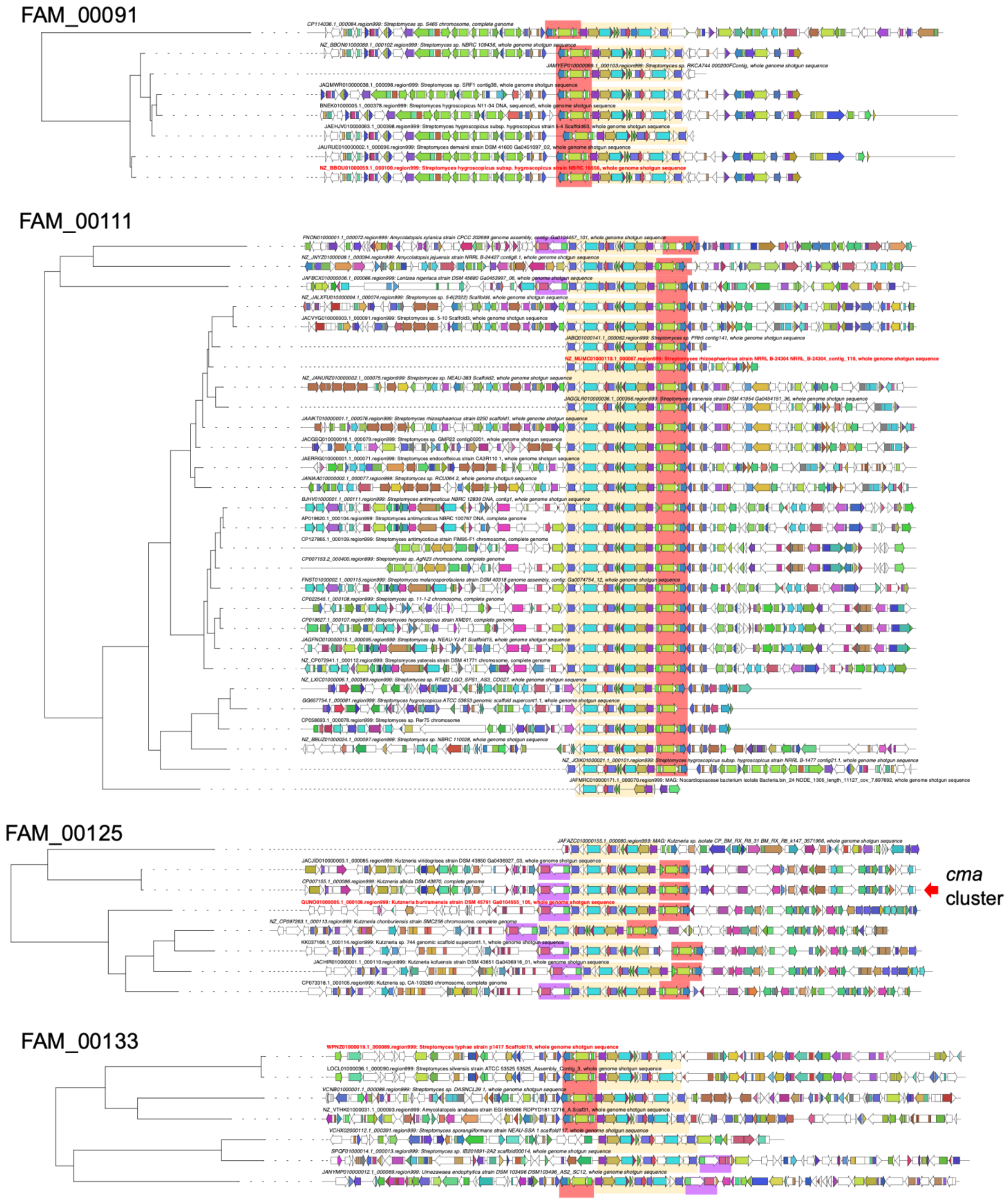

Figure S2. The BiG-SCAPE analysis of *ava*-like clusters (Part 2). The clusters belong to FAM_00091, FAM_00111, FAM_00125, and FAM_00133 are shown. Most of these clusters do not have *avaA8* and *avaC* homologs but have a *cmaG*-like gene encoding an FMN-dependent oxidoreductase and a *cmaR*-like gene encoding a LysR family transcriptional regulator. *avaI*, *H*, *A1, A2, A3, A4, A5*, *A6*, *B*, and *A7* homologs are highlighted in yellow. *avaE* and *avaD* homologs are highlighted in purple. *cmaG* and *cmaR* homologs are highlighted in red. The colors of Pfam domains are automatically generated by BiG-SCAPE.

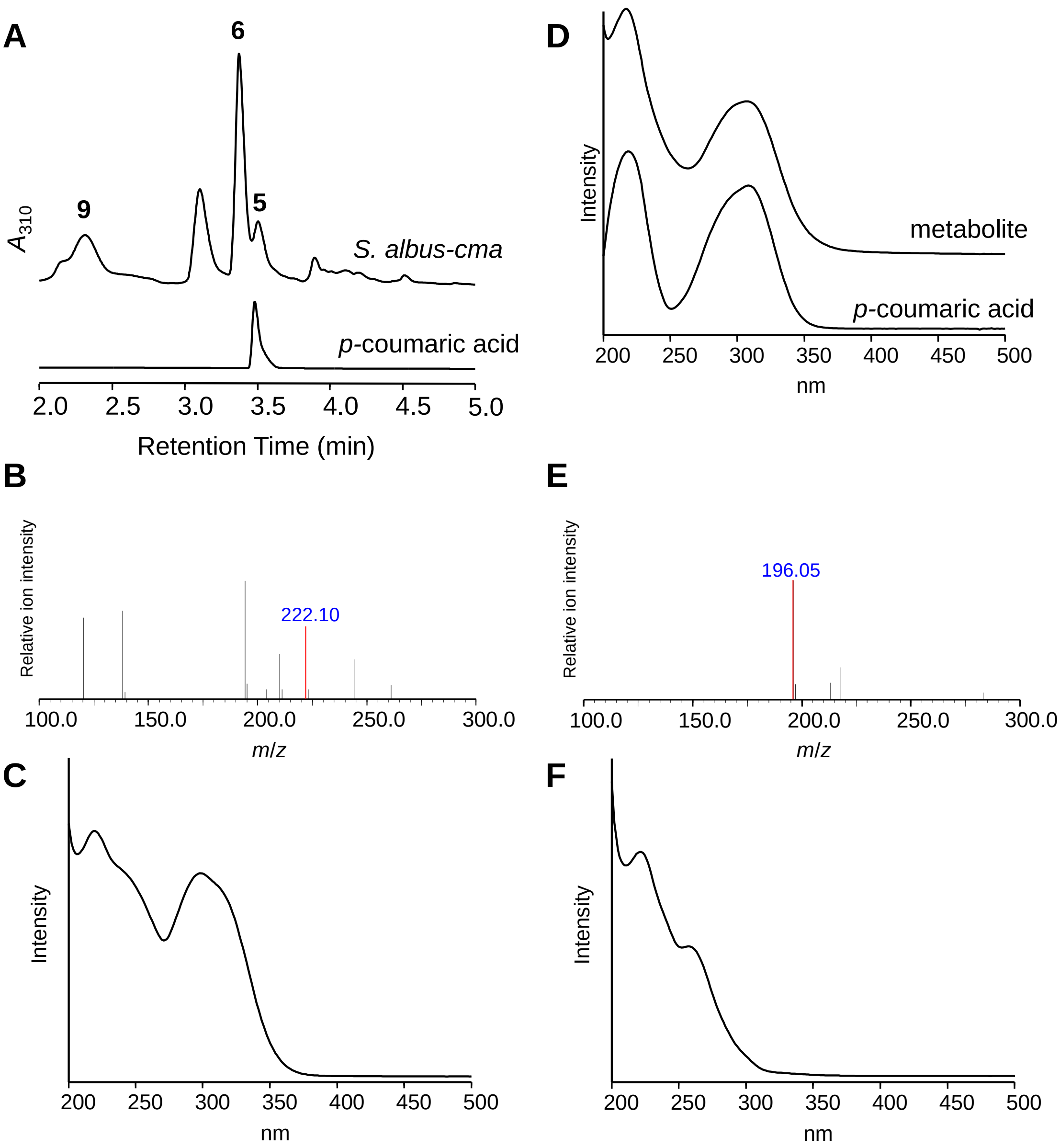

Figure S3. LC-MS analysis of the metabolites of *S. albus-cma*, which produces compounds **5** and **6** and other 3,4-AHBA derivatives. (**A**) Comparison of compound **5** from the metabolites of *S. albus-cma* and authentic *p*-coumaric acid. UV chromatograms at 310 nm are shown. (**B**) Mass spectra of compound **6** from the metabolites of *S. albus-cma*. (**C**) UV spectrum of the compound **6**. (**D**) Comparison of the UV spectra of compound **5** from the metabolites of *S. albus-cma* and authentic *p*-coumaric acid. (**E**) Mass spectra of compound **9** from the metabolites of *S. albus-cma*. $[M + H]^+$ ion at m/z = 196 corresponds to that of *N*-acetyl-3,4-AHBA. (**F**) UV spectrum of compound **9**.

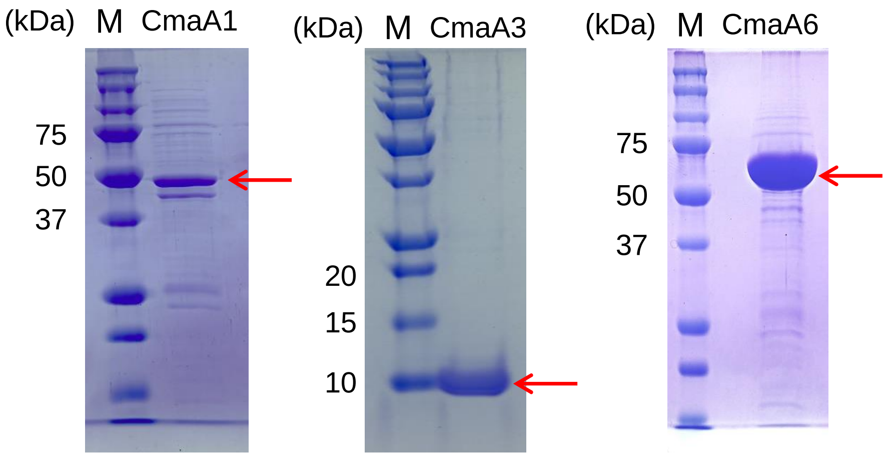

Figure S4. SDS-PAGE analysis of the recombinant proteins used in this study. All recombinant proteins were produced by *E. coli* BL21(DE3). The theoretical molecular mass values (kDa) of recombinant proteins are CmaA1, 50.6; CmaA3, 10.5; and CmaA6, 62.0.

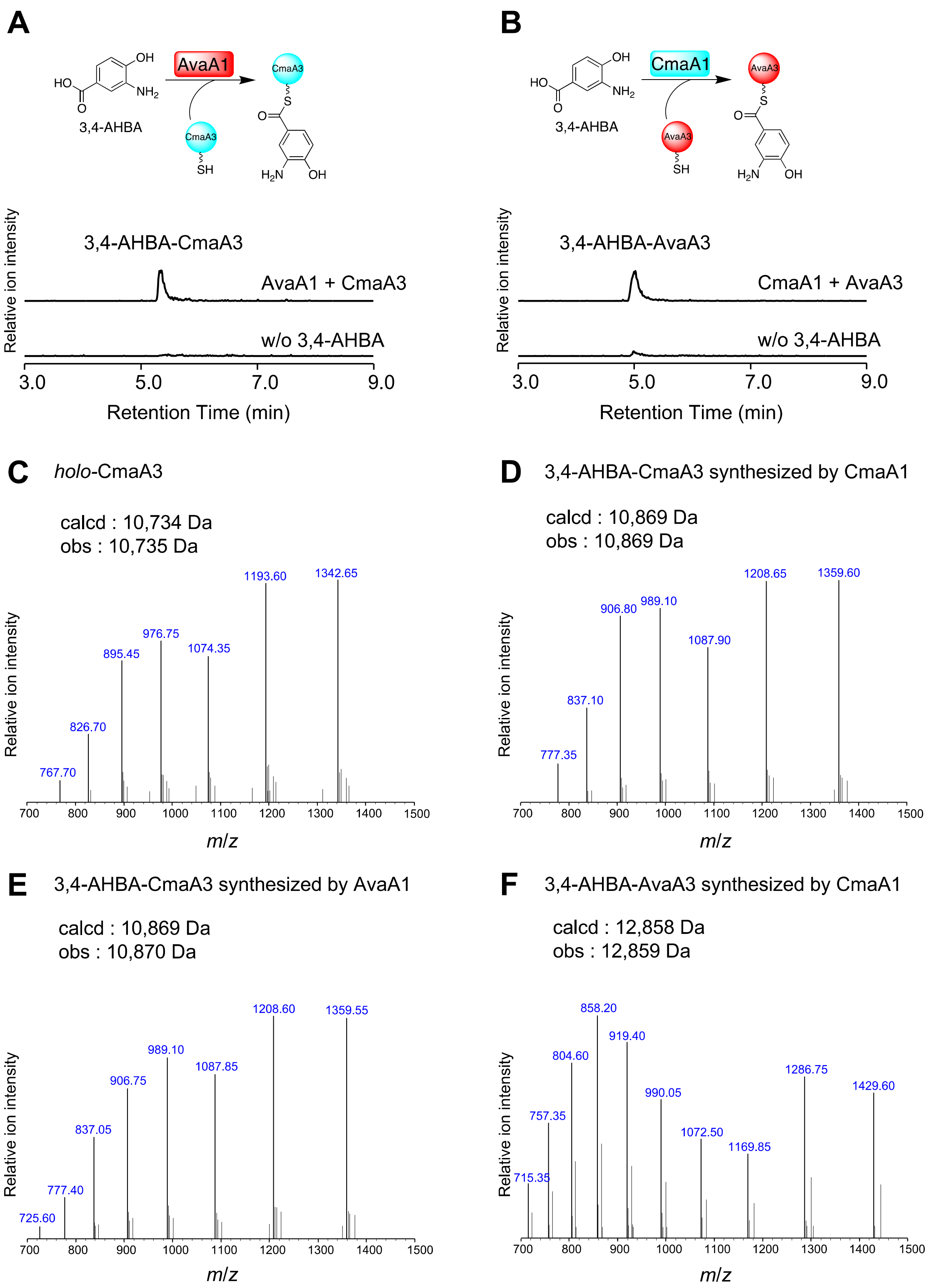

Figure S5. *In vitro* analysis of CmaA1, *holo*-CmaA3, AvaA1, and *holo*-AvaA3. (**A**) 3,4-AHBA was loaded onto *holo*-CmaA3 by AvaA1. Extracted ion chromatograms of *m/z* = 1087.9, which corresponds to [M + 10H]$^{10+}$ of 3,4-AHBA-CmaA3 under positive ion mode, are shown. (**B**) 3,4-AHBA was loaded onto *holo*-AvaA3 by CmaA1. Extracted ion chromatograms of *m/z* = 1287.0, which corresponds to [M + 10H]$^{10+}$ of 3,4-AHBA-AvaA3 under positive ion mode, are shown. (**C-F**) The mass spectra shift of *holo*-CmaA3 (**C**), 3,4-AHBA-CmaA3 synthesized by CmaA1 (**D**), 3,4-AHBA-CmaA3 synthesized by AvaA1 (**E**), and 3,4-AHBA-AvaA3 synthesized by CmaA1 (**F**).

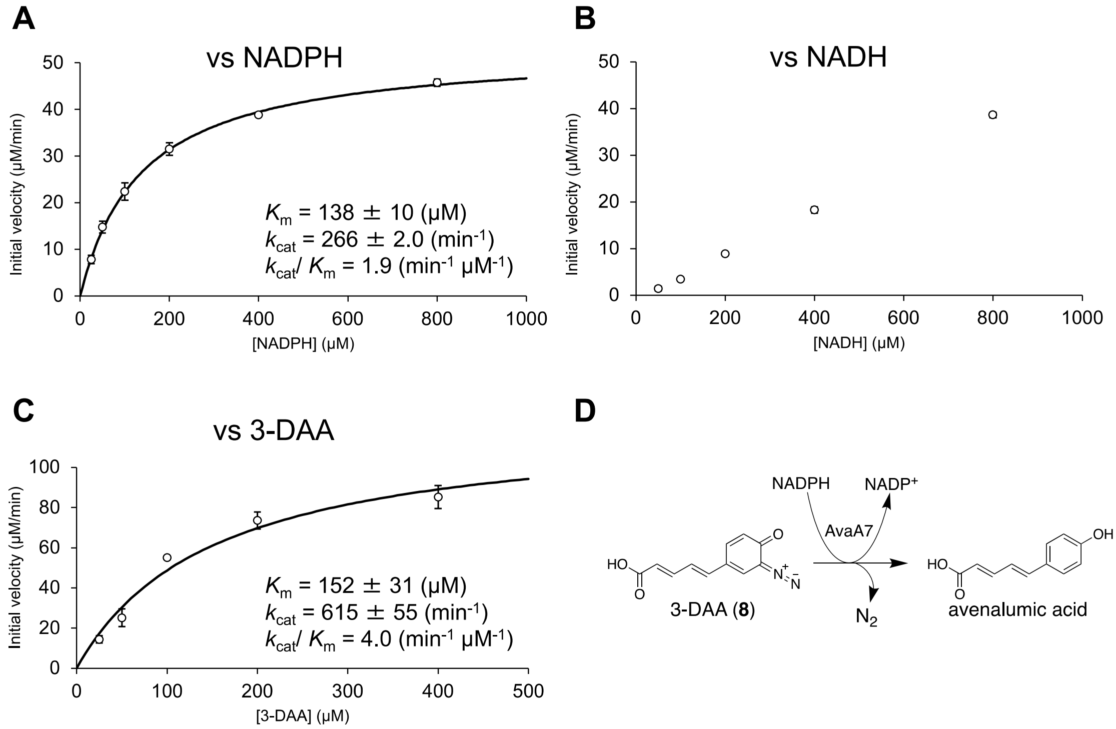

Figure S6. Kinetic analysis of AvaA7, fitted with the Michaelis-Menten equation. The error bars represent the standard error (n = 3). (**A**) Kinetic analysis for NADPH. (**B**) Kinetic analysis for NADH, which could not be fitted by the Michaelis-Menten equation probably because of the high K_m value. (**C**) Kinetic analysis for 3-DAA (**8**) when NADPH was used as a cofactor. (**D**) The schematic representation of the reaction focused on this experiment.

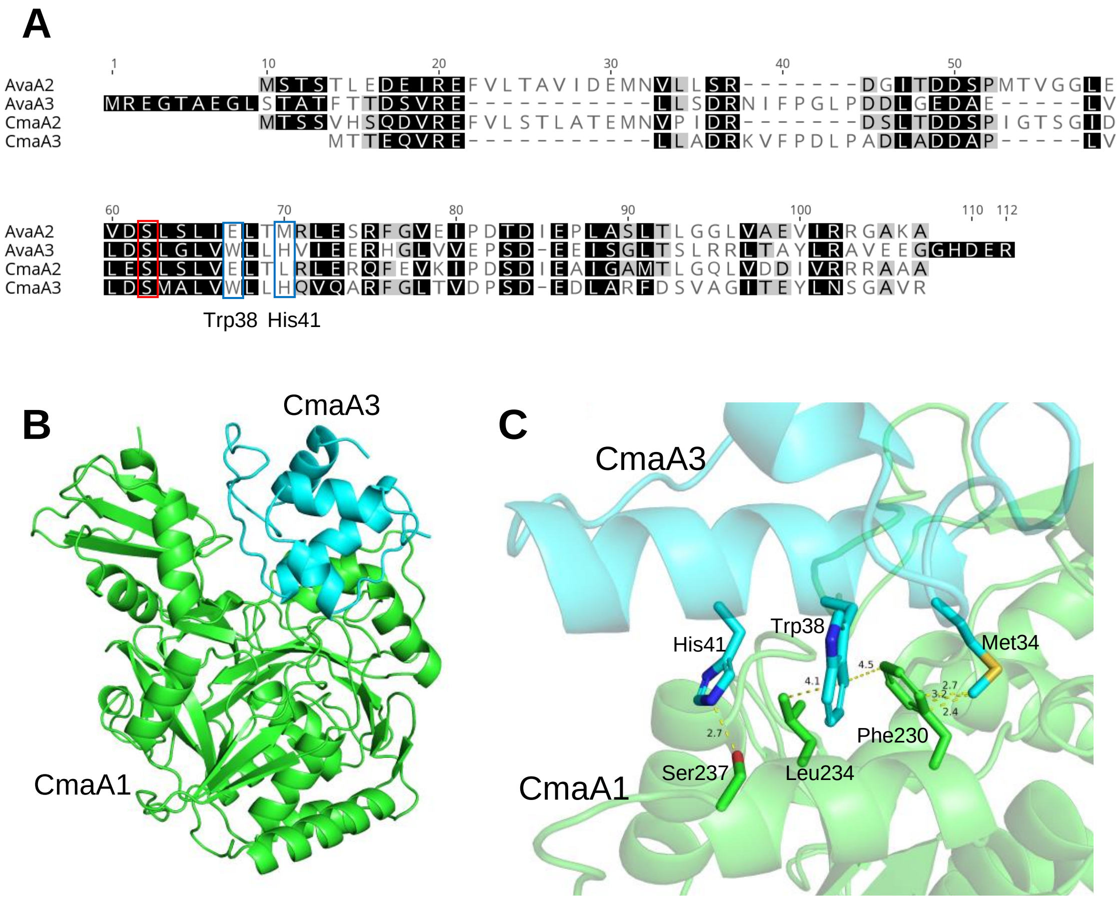

Figure S7. Insights into the mechanism of partner ACP (CmaA3) recognition mechanism by CmaA1. (**A**) Amino acid alignment of AvaA2, AvaA3, CmaA2, and CmaA3. The Ser residues to which the phosphopantetheinyl arm binds are labeled with a red box. Residues labeled with blue boxes are predicted to be important for the partner ACP recognition by AvaA1 and CmaA1. (**B**) The structure model of CmaA1-CmaA3 complex predicted by AlphaFold2 [1]. CmaA1, green; CmaA3, cyan. (**C**) Enlarged view of the interface between CmaA1 and CmaA3. Met34, Trp38, and His41 in CmaA3 seem to be important for the interaction. Trp38 and His41 are conserved between AvaA3 and CmaA3.

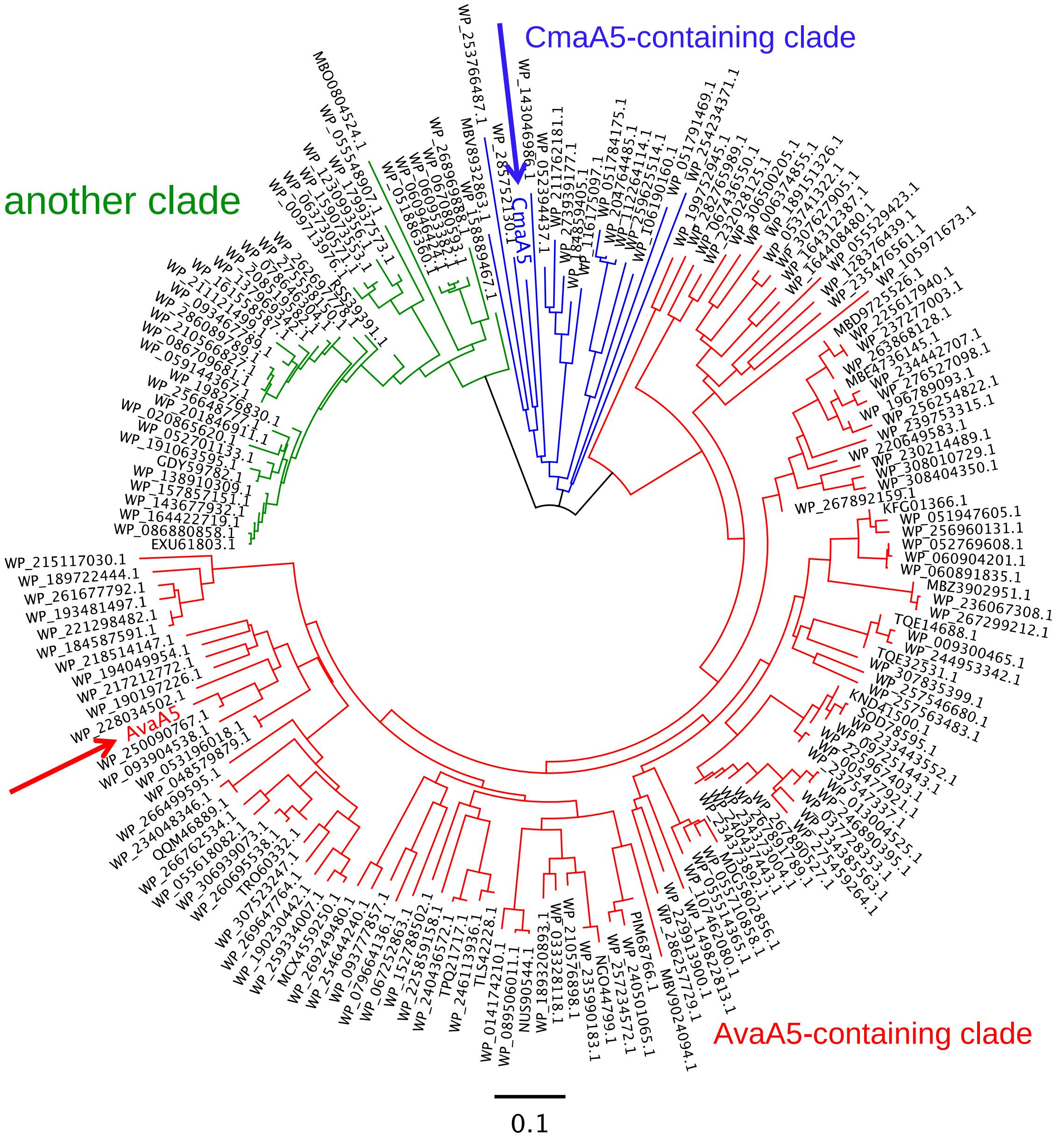

Figure S8. Phylogenetic analysis of CLFs encoded by the by *ava*-related BGCs in the database. These enzymes are divided into three large clades. AvaA5 and CmaA5 are marked with arrows. The distance between each enzyme was determined by the global alignment using BLOSUM62.

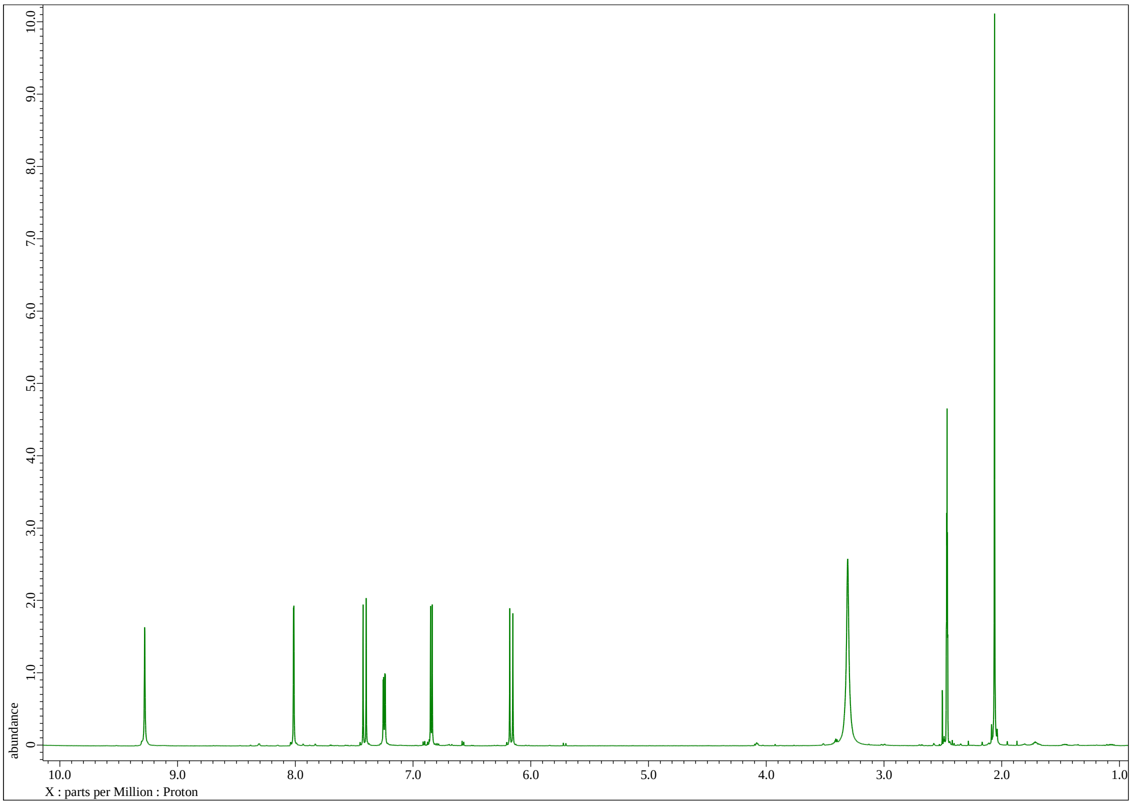

Figure S9. ^{1}H NMR spectrum of compound **6**.

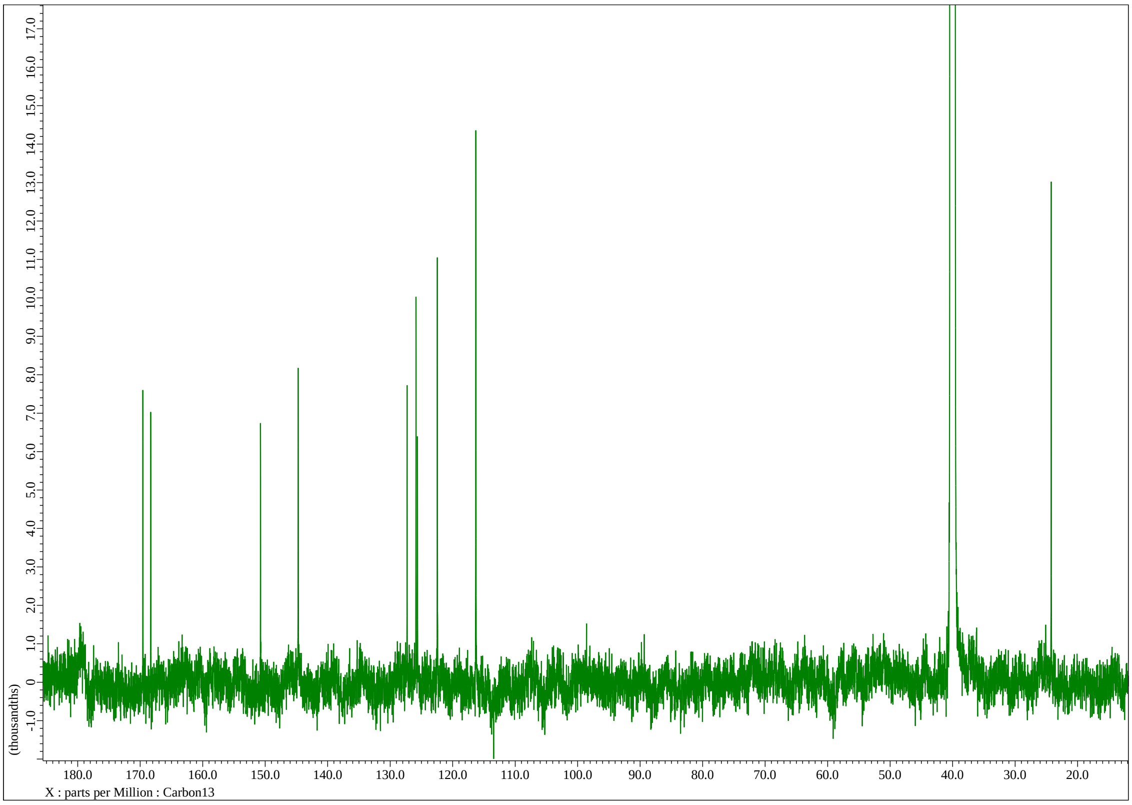

Figure S10. ^{13}C NMR spectrum of compound **6**.

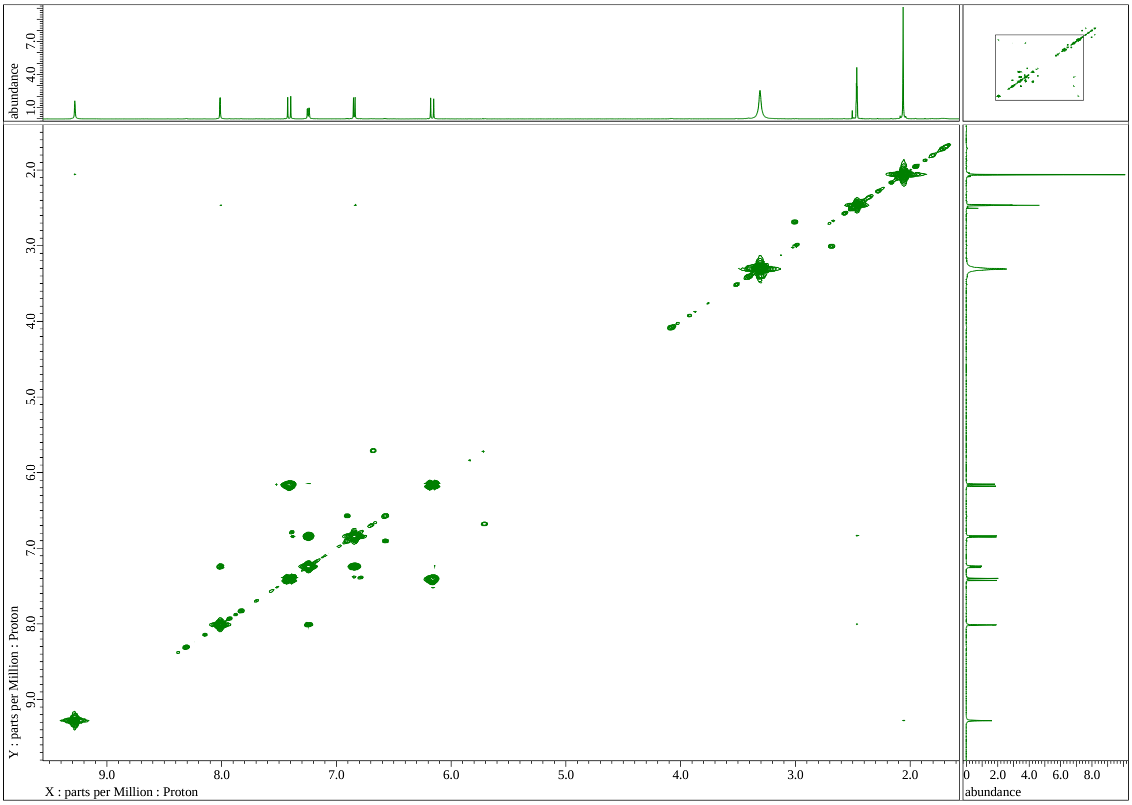

Figure S11. $^{1}H,^{1}H$ COSY spectrum of compound **6**.

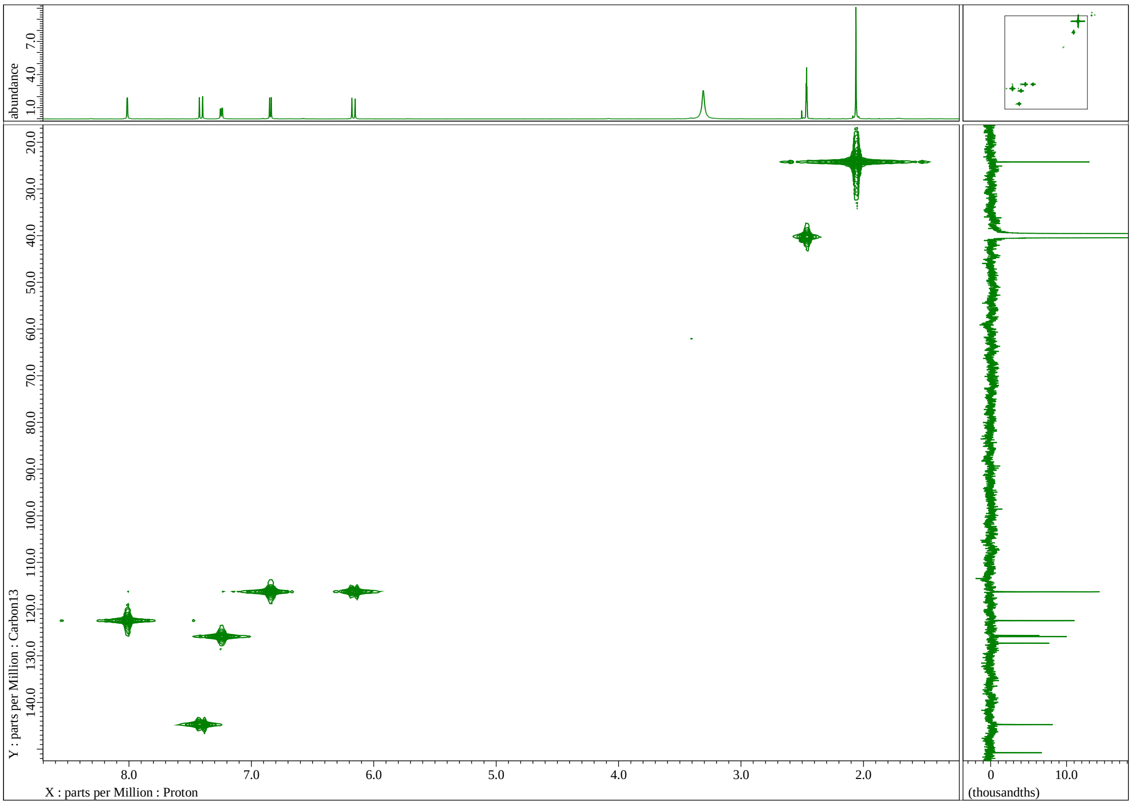

Figure S12. $^{1}H,^{13}C$ HMQC spectrum of compound **6**.

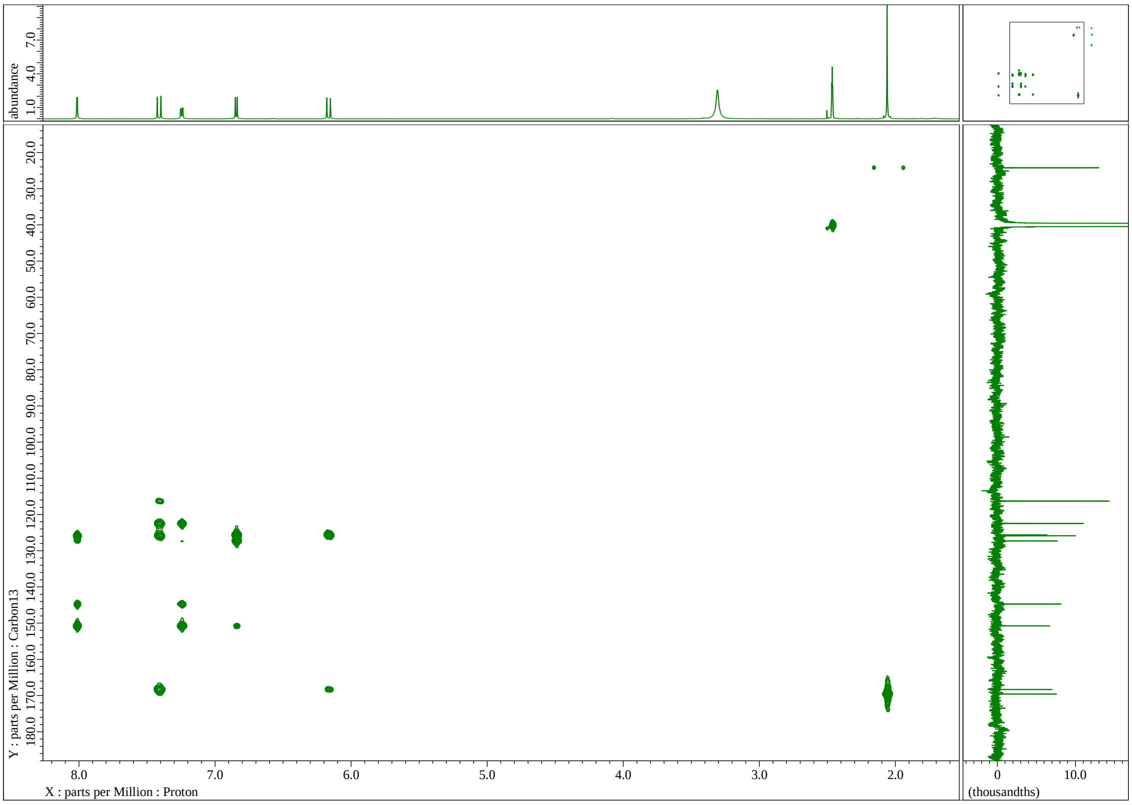

Figure S13. $^{1}H,^{13}C$ HMBC spectrum of compound **6**.

References

[1] J. Jumper, R. Evans, A. Pritzel, T. Green, M. Figurnov, O. Ronneberger, K. Tunyasuvunakool, R. Bates, A. Žídek, A. Potapenko, A. Bridgland, C. Meyer, S. A. A. Kohl, A. J. Ballard, A. Cowie, B. Romera-Paredes, S. Nikolov, R. Jain, J. Adler, T. Back, S. Petersen, D. Reiman, E. Clancy, M. Zielinski, M. Steinegger, M. Pacholska, T. Berghammer, S. Bodenstein, D. Silver, O. Vinyals, A. W. Senior, K. Kavukcuoglu, P. Kohli, D. Hassabis, *Nature* **2021,** *596*, 583–589.
